# A Rare Cause of Type II Neovascularization: Unilateral Retinal Pigment Epithelium Dysgenesis

**DOI:** 10.4274/tjo.galenos.2020.89814

**Published:** 2020-06-27

**Authors:** Berrak Şekeryapan Gediz

**Affiliations:** 1 University of Health Sciences Turkey, Ankara Ulucanlar Eye Training and Research Hospital, Ankara, Turkey

**Keywords:** Unilateral retinal pigment epithelium dysgenesis, type II neovascularization, bevacizumab

## Abstract

Unilateral retinal pigment epithelium dysgenesis (URPED) is a very rare clinical condition first described in 2002. Fundus examination and imaging findings are almost pathognomonic and can facilitate diagnosis of this uncommon disease. In this article, we present a 32-year-old patient who developed type II neovascularization (NV) as a complication of URPED. After 6 months of monthly intravitreal bevacizumab injection, visual acuity increased from 20/32 to 20/20 but optic coherence tomography findings were partially improved. The aim of this report is to highlight URPED and secondary type 2 NV, the pathogenesis and prognosis of which are unknown but which cause visual loss especially in the younger population.

## Introduction

Unilateral retinal pigment epithelium dysgenesis (URPED) is a very rare, unilateral condition that affects the younger population. It is typically characterized by a single leopard-spot lesion with a seashell-like scalloped appearance located in the posterior pole and extending to the optic nerve. The lesion is in the RPE layer and gets its leopard-spot appearance due to fibrotic and hyperplastic changes in its periphery and areas thinning in its center. Diagnosis is established with fundoscopic examination together with fluorescein angiography (FA) and fundus autofluorescence (FAF), which provide reverse images.^1,2^ Visual prognosis depends on the presence of associated neovascularization (NV) (type 1: choroidal NV, type II: subretinal NV, type III: retinal angiomatosis proliferation).^2,3,4^

In this report, we present the treatment of a patient with type II NV secondary to URPED with intravitreal bevacizumab. Our aim was to highlight URPED and secondary NV, which is extremely rare but causes vision loss in the younger population.

## Case Report

**Figure 1:**
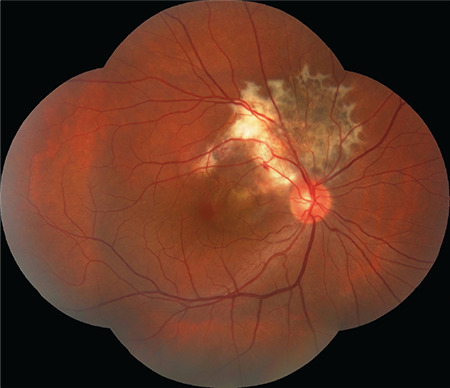
Color fundus photographs of the right eye show an RPE lesion with well-defined margins and a seashell-like scalloped appearance. Leopard-spot pattern is observed in the part of the lesion superior to the superior temporal arcade and a large subretinal scar formation is observed in the part of the lesion inferior to the superior temporal arcade. Retinal folds are noted in the macula and fovea is raised

**Figure 2:**
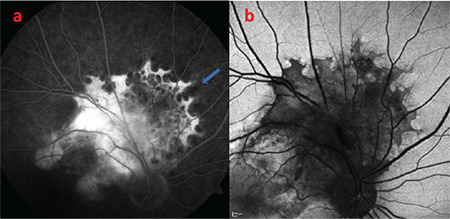
a) Fluorescein angiography demonstrates well-defined hyperfluorescent margins in the part of the lesion superior to superior temporal arcade, surrounded by dark ovals. b) Fundus autofluorescence shows the lesion and its margins are hypoautofluorescent, giving a reverse image of fluorescein angiography

**Figure 3:**
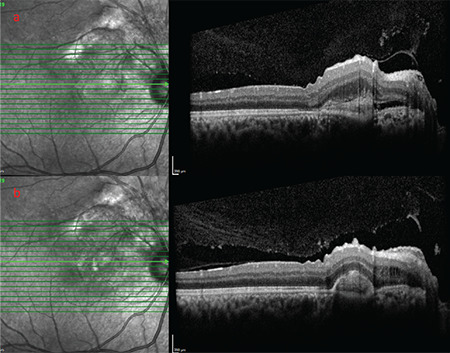
a) OCT cross-section of the left eye passing through fovea demonstrated type 2 NV, subretinal fluid, irregularity of the retinal surface, and thickening of the retina over the NV prior to treatment. b) OCT image of the same eye after 6 months of treatment shows regression of the subretinal fluid but persistent intraretinal fluid

A 32-year-old man presented with blurry vision in his right eye. He had no known diseases or history of trauma. Best corrected visual acuity (BCVA) was 20/32 in the right and 20/20 in the left eye. Intraocular pressure was 14 mmHg in the right eye and 12 mmHg in the left eye; anterior segment examination results were normal. Fundus examination revealed a lesion with well-defined, scalloped margins that extending from the right peripapillary region to the macula and superior quadrant, including the superior temporal vascular arcade. The part of the lesion superior to the superior temporal arcade exhibited the leopard-spot pattern while a large subretinal scar formation was observed in the part of the lesion inferior to the superior temporal arcade, and the fovea was raised. Retinal folds were visible in the macula. The vessels superior to the optic nerve appeared thin and lacked continuity (Figure 1). On FA, the lesion was generally hyperfluorescent; the part of the lesion superior to the superior temporal arcuate had very distinct hyperfluorescent edges surrounded by dark ovals (Figure 2a). The lesion and its margins appeared hypoautofluorescent on FAF imaging (Figure 2b). The optic coherence tomography (OCT) cross-section passing through the fovea demonstrated type II NV, subretinal fluid, retinal surface irregularity, and thickening of the retina over the NV (Figure 3a). Type II NV secondary to URPED was diagnosed and intravitreal bevacizumab (IVB) (1.25 mg/0.05 mL) therapy was initiated. At 1-month follow-up, the patient’s BCVA had decreased to 20/32 and there were no changes in the OCT findings. After 6 monthly IVB injections, BCVA improved to 20/20 and OCT showed regression of the subretinal fluid but persistent intraretinal fluid (Figure 3b). The patient is continuing IVB therapy.

## Discussion

Dysgenesis refers to the abnormal or defective development of an organ. URPED is an extremely rare clinical condition of unknown etiopathogenesis, of which only 20 cases have been reported in the literature to date. It was first described by Cohen et al.^1^ in 2002 in 4 patients with unilateral, idiopathic, leopard-spot lesions of the RPE, 2 of whom also had choroidal NV. In 2009, they named the lesion URPED and with the addition of the previous 4 patients, presented the clinical characteristics of a total of 9 cases.^2^ Retinal symptoms associated with URPED include epiretinal membrane, increased retinal vascular tortuosity, and retinal folds. Shimoyama et al.^3^ published a case of choroidal NV secondary to URPED in 2014 and reported that the lesion did not respond to 2 doses of subTenon’s triamcinolone acetonide and 1 dose of IVB injection. In 2019, Preziosa et al.^4^ reported a case of choroidal NV secondary to URPED in which they attained both functional and anatomical success after 2 doses of IVB. The type 2 NV lesion in our case responded slowly to IVB therapy and 6 months of monthly injections resulted in complete recovery of visual acuity but did not fully inactivate the lesion.

Despite its typical clinical appearance, URPED is most commonly confused with combined hamartoma of the retina and RPE. This lesion is also a rare clinical condition and is characterized by retinal thickening, epiretinal membrane, and vascular tortuosity.^5^ There is one publication reporting that URPED may be an atypical form of combined hamartoma of the retina and RPE.^6^ However, they can be distinguished based on FA and FAF imaging, which is pathognomonic for URPED, and clinical findings.

Traumatic retinopathy is a clinical condition that is included in the differential diagnosis of URPED. Acute contusion necrosis, also known as commotio retinae, and resolution of hemorrhagic retinal detachment may lead to a similar appearance.^7^

Although the visual prognosis of URPED is not clear, it has been shown to slowly progress toward fovea over a period of years and cause serious vision loss.^8^ Moreover, the NV that develops as a complication impacts visual prognosis in URPED patients. Although there is insufficient information in the literature to reach a definite conclusion, it should be kept in mind based on the present case that NV lesions respond slowly to IVB therapy.
